# Very small size proteoliposomes abrogate cross-presentation of tumor antigens by myeloid-derived suppressor cells and induce their differentiation to dendritic cells

**DOI:** 10.1186/2051-1426-2-5

**Published:** 2014-03-11

**Authors:** Audry Fernández, Liliana Oliver, Rydell Alvarez, Arletty Hernández, Judith Raymond, Luis E Fernández, Circe Mesa

**Affiliations:** 1 Immunobiology Division, Center of Molecular Immunology, 216 St and 15th Ave., Atabey, Playa, P.O. Box 16040, Havana 11600, Cuba; 2 Systems Biology Division, Center of Molecular Immunology, 216 St and 15th Ave., Atabey, Playa, P.O. Box 16040, Havana 11600, Cuba; 3 Innovation Division, Center of Molecular Immunology, 216 St and 15th Ave., Atabey, Playa, P.O. Box 16040, Havana 11600, Cuba

**Keywords:** Adjuvants, MDSCs, Tumors, Cross-presentation, APCs

## Abstract

**Background:**

Myeloid-derived suppressor cells (MDSCs) are among the major obstacles that adjuvants for cancer vaccines have to overcome. These cells cross-present tumor-associated antigens (TAA) to naive T lymphocytes with a tolerogenic outcome. Very Small Size Proteoliposomes (VSSP) is used as adjuvant by four therapeutic cancer vaccines currently in Phase I and II clinical trials. We previously found that VSSP reduces the suppressive function of MDSCs, then activating antigen-specific CTL responses in tumor-bearing (TB) mice, with the consequent reduction of tumor growth. However the mechanistic explanation for the immunomodulatory effect of this adjuvant in TB hosts has not been addressed before.

**Methods:**

TB mice were inoculated with VSSP and MDSCs isolated and characterized by their expression of *Arg1* and *Nos2* genes by RT-PCR. The effect of VSSP on antigen cross-presentation by MDSCs, regulatory T cells (Tregs) expansion and MDSCs differentiation towards dendritic cells (DCs) was analyzed by FACS. Student’s t test or ANOVA and Tukey’s tests were used for statistical analyses.

**Results:**

After inoculating VSSP into TB mice, a significant reduction of *Arg1* and *Nos2* gene expression was observed in recovered MDSCs. Concurrently the ability of these cells to induce down-regulation of CD3ζ chain on T cells was lost. Likewise in mice inoculated with the adjuvant lower percentages of Tregs were detected. *In vitro,* VSSP treatment was enough to differentiate MDSCs into phenotypically mature DCs, eliminating the former suppressive effect. Noteworthy, *in vivo* administration of VSSP to OVA-expressing (EG.7) TB mice abrogated this model antigen cross-presentation by splenic MDSCs. Similar results were obtained even when OVA antigen was administered into these TB mice formulated in VSSP. On the contrary, immunization with the same protein in polyI:C did not change the percentage of MDSCs expressing SIINFEKL/H-2K^b^ complexes, whereas a concomitant injection of VSSP aborted the limitations of polyI:C in this setting.

**Conclusions:**

Altogether, these results indicate that VSSP has the peculiar capacity of inhibiting TAA cross-presentation and certain suppressive mechanisms on MDSCs which in turn, combined with the ability to induce differentiation of these cells into antigen-presenting cells (APCs), sustains this adjuvant as an ideal immunomodulator for cancer immunotherapy.

## Background

The induction of an effective CTL activity is difficult to achieve in the immunosuppressive environment of a tumor. Tregs, MDSCs and tumor-associated macrophages have been recognized as the main inhibitory populations recruited by tumors with relevant contribution to the dampening of antitumor immune response [1-3].

Particularly, MDSCs are a heterogeneous population of myeloid origin, which express CD11b and Gr1 markers in mice [4] but their phenotype in human cancers is rather diverse [5,6]. MDSCs-mediated suppression of CD8^+^ T cell [7-9] and NK cell [10,11] functions has been well characterized. In contrast, the inhibition of CD4^+^ T cell responses remained elusive until a recent work of Nagaraj *et al.* in which the authors demonstrate that antigen-specific CD4^+^ T cells are suppressed only by MCH II-expressing MDSCs [12]. Although the mechanisms of MDSCs-mediated suppression are quite diverse, there is a general acceptance of the important role of L-arginine metabolizing enzymes, arginase (ARG) and nitric oxide synthase (NOS), with the Nox family of phagocytic oxidases [4,13,14]. It has been shown that splenic MDSCs can cross-present tumor antigens to CD8^+^ T cells, which leads to tolerance induction [15]. Furthermore, the immunosuppressive network associated to cancer is reinforced by MDSCs that not only expand Tregs [16,17] but can also differentiate into tumor-associated macrophages within the tumor microenvironment [18,19].

Considering this complex scenario, antitumor immunotherapy requires not only of relevant antigens but also of suitable immunomodulators to overcome tumor-induced immunosuppression. Compounds like docetaxel, all-trans retinoic acid and synthetic oligodeoxynucleotides containing unmethylated CpG motifs (CpG ODN) accelerate the differentiation of MDSCs into mature leukocytes [20-23]. Moreover, some adjuvants are able to reduce the inhibitory function of tumor-induced MDSCs [23-25]. Among these, we have previously reported the VSSP, which is a nanoparticulated adjuvant obtained by the combination of outer membrane vesicles from *Neisseria meningitidis* (containing TLR2 and TLR4 agonists) and GM3 ganglioside [26]. This adjuvant induces DCs maturation and antigen cross-presentation to CD8^+^ T cells in tumor-free mice [27,28]. More recently, we demonstrated that VSSP protects CTL responses specific for the nominal antigen not only in TB mice but also in the context of severe leukopenia [24,29]. Currently four therapeutic cancer vaccines using this product as adjuvant are in clinical research. Two of the formulations, based on the epidermal growth factor receptor [30] and the vascular endothelial growth factor [31] recombinant proteins, are in Phase I clinical trials. Other two candidates, a human papilloma virus peptide vaccine [32] and a gonadotropin releasing hormone-based vaccine [33], have already finished their safety and immunogenicity studies and are currently being tested in Phase II trials in women with high-grade cervical intraepithelial neoplasia and in prostate tumor patients, respectively [34]. Moreover, an ongoing physician-lead trial in patients with metastatic renal cell carcinoma intends to evaluate the effect of VSSP on MDSCs-mediated immunosuppression.

The aim of the present research was to assess the influence of VSSP on the classical suppressive mechanisms of MDSCs. A significant reduction in the expression of *Arg1* and *Nos2* genes was observed as a consequence of VSSP inoculation. This could be related with the observation that MDSCs from TB mice injected with the adjuvant lost their ability to down-regulate CD3ζ chain on T cells. Simultaneously, we detected a lower percentage of Tregs in these mice as well as a higher expression of CD62L on T lymphocytes. Of interest was the observation that VSSP induced differentiation of MDSCs towards DCs expressing maturation markers, but the most surprising result was the inhibition caused by VSSP on cross-presentation of TAA by splenic MDSCs. Our results suggest that VSSP could be used during immunotherapy to modulate MDSCs-mediated suppression of antitumor response, including the expansion of Tregs.

## Results

### VSSP administration into TB mice potentiates CTL responses and skews Th differentiation patterns

**Figure 1:**
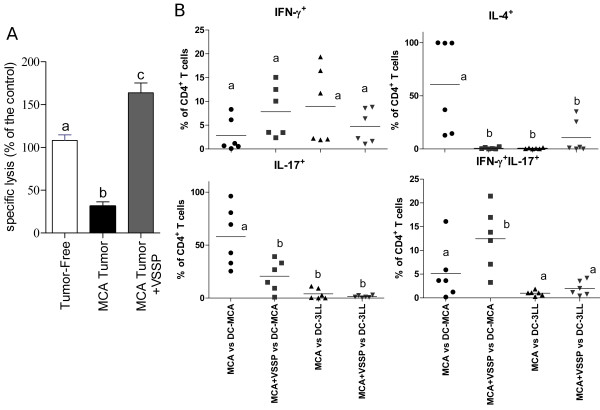
**VSSP modulates Th differentiation and protects CTL responses in TB mice.** Mice bearing MCA203 tumors were s.c. inoculated with three doses of VSSP, or left untreated. **(A)** Mice were also vaccinated with OVA protein mixed with polyI:C and *in vivo* CTL response was measured. Graph shows the specific lysis, as a percentage of the control (tumor-free mice). Data are represented as mean of three individual mice ± SD. Two experiments with similar results were done. **(B)** Splenocytes from MCA203 TB mice, treated or not with VSSP, were stimulated *in vitro* with BM-DCs, previously pulsed with tumor lysates obtained from MCA203 and 3LL-D122 tumor cell lines. Production of IL-4, IL-17 and IFN-γ was detected by intracellular staining after 96 h stimulation. Graphs indicate the percentage of cells producing each cytokine, within the CD4^+^ T cells gate, for six individual mice belonging to two different experiments. Statistical analyses were performed with ANOVA and Tukey’s tests **(A)** or Kruskal-Wallis and Dunn’s tests **(B)**.

We have previously shown that VSSP administration into MCA203 TB mice significantly reduces these tumors’ growth [24]. To understand the mechanisms associated with the antitumor effect of VSSP, we began by studying the effector T cell responses in these mice. MCA203 TB mice displayed an impaired CTL response to OVA, formulated with polyI:C as adjuvant, relative to vaccinated tumor-free mice (Figure 1A and Additional file 1: Figure S1A). Interestingly, when TB mice injected with OVA/polyI:C additionally received three doses of VSSP alone, the detected antigen-specific CTL response was significantly higher than in TB mice non-treated with the adjuvant and even superior than in tumor-free mice vaccinated with OVA/polyI:C (Figure 1A and Additional file 1: Figure S1A).

Considering that VSSP is a Th1-prone adjuvant, we examined whether it was able to change the tumor-induced polarization of TAA-specific Th cells (Additional file 1: Figure S1B). Splenocytes from VSSP-treated or untreated MCA203 TB mice were *in vitro* stimulated with bone marrow (BM)-derived DCs pulsed with tumor lysates, obtained from MCA203 or 3LL-D122 (negative control) cell lines, and the production of IFN-γ, IL-4 and IL-17 by CD4^+^ T cells was measured by intracellular staining. As shown in Figure 1B, TAA-specific Th cells from non-treated TB mice produced mainly IL-4 or IL-17, whereas in VSSP-treated TB mice IL-4^+^ Th cells were undetectable. A significant reduction in the production of IL-17 by Th cells was also observed in VSSP-treated TB mice, associated with an increase in IFN-γ/IL-17 double positive cells (Figure 1B).

**Figure 2:**
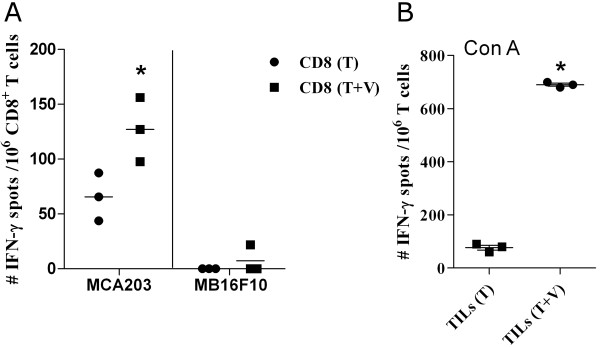
**Effector functions of TILs are improved as a consequence of VSSP inoculation.** MCA203 TB mice were inoculated with VSSP on days 11, 12 and 18. On day 22, the s.c. tumors were extracted and TILs were isolated. **(A)** Tumor-specific response of CD8^+^ TILs after stimulation for 72 h with the MCA203 tumor cell line, or MB16F10 negative control. IFN-γ production by 10^6^ CD8^+^ TILs was evaluated with ELISPOT assay. **(B)** Non-specific stimulation of TILs with Con A mitogen was additionally performed and the IFN-γ production was also evaluated by ELISPOT assay. Graphs indicate the mean of the number of IFN-γ spots from one experiment representative of two (T: tumor and V: VSSP). Statistically significant differences were detected with Student’s t test.

Likewise, changes in the functionality of tumor-infiltrating lymphocytes (TILs) derived from MCA203 TB mice, inoculated or not with VSSP, were evaluated. As shown in Figure 2A, a higher frequency of IFN-γ secreting CD8^+^ TILs was detected in VSSP-treated TB mice after specific *in vitro* stimulation with the MCA203 tumor cell line. As expected, no CD8^+^ T cell response was observed under stimulation with the non-related MB16F10 tumor cell line, indicating that VSSP potentiated tumor-specific CD8^+^ T cell activation. In addition, VSSP reduced the functional impairment caused by MCA203 tumor on TILs, as demonstrated through the mitogenic stimulation with Con A (Figure 2B). Overall, the interference with tumor-induced suppression of CD4^+^ and CD8^+^ T cell responses, due to VSSP administration, could be contributing to the antitumor activity of this adjuvant in MCA203 TB mice.

### Tumor-promoted recruitment of Tregs is impaired by VSSP even in a context of increased numbers of MDSCs

It has been formerly demonstrated that MCA203 sarcomas induce the accumulation of suppressive MDSCs in the spleen and tumor microenvironment [7,24]. Presently, a corroboration of our previous results [24] demonstrating that mice bearing MCA203 tumors injected with VSSP (Additional file 1: Figure S1B) almost duplicate splenic CD11b^+^Gr1^+^ cells, compared to untreated TB mice, was achieved (data not shown). However, CD11b^+^Gr1^+^ cells isolated from these VSSP-inoculated TB mice were phenotypically similar to those from VSSP-injected tumor-free mice and different from untreated tumor-induced counterparts (Additional file 2: Figure S2A).

**Figure 3:**
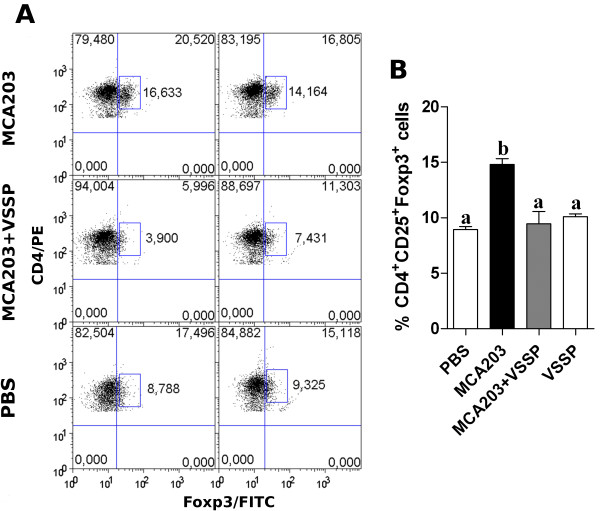
**VSSP inhibits tumor-mediated expansion of Tregs in mice with high percentages of MDSCs.** Splenic CD4^+^CD25^+^Foxp3^+^ Tregs were analyzed by FACS in VSSP-inoculated MCA203 TB mice, in comparison to untreated TB mice or tumor-free mice. A group of tumor-free mice inoculated with VSSP was also studied. Tregs percentages are referred to the gate of CD4^+^ T cells. **(A)** Dot plot graphs show the staining of Tregs in two representative mice per group. **(B)** Data are expressed as mean ± SD of five individual mice per group. ANOVA and Tukey’s tests were applied for statistical comparison. Data are representative of two experiments with similar results.

Looking at Tregs frequency, as expected from the literature, an increase of splenic CD4^+^CD25^+^ T cells expressing Foxp3 was detected, from 8.75 ± 0.80% in tumor-free control mice to 14.80 ± 1.17% in MCA203 TB mice (Figure 3A and B), connecting with one of the proposed mechanisms for MDSCs mediated-suppression (i.e. the expansion of Tregs that reinforce the suppression of tumor-specific responses) [16,17]. Remarkably, in VSSP-treated TB mice, in which the percentages of CD11b^+^Gr1^+^ cells were higher than in untreated TB mice, we found a significant reduction in the percentages of splenic Tregs (Figure 3A and B). In fact, no difference in Tregs frequencies was observed between TB mice inoculated with VSSP and tumor-free control mice. Additionally, the inoculation of VSSP in tumor-free mice did not modify the normal percentages of Tregs (Figure 3B), suggesting that this adjuvant does not have a direct effect on Tregs *in vivo*.

### Interference of VSSP with the classical suppressive mechanisms of MDSCs

Our previous results indicate that VSSP administration to MCA203 TB mice reduces the suppression exerted by tumor-induced splenic MDSCs on CD8^+^ T cells specific for TAA [24]. Similarly, IFN-γ release and lytic activity of OVA-specific CD8^+^ T cells were significantly inhibited by MDSCs isolated from MCA203 TB mice, whereas CD11b^+^Gr1^+^ cells obtained from VSSP-treated counterparts were non suppressive (Additional file 2: Figure S2B and C). To increase our understanding of the effect of VSSP on tumor-induced MDSCs, certain classical mechanisms associated with the suppressive function of these cells were addressed following the schedule depicted in Additional file 1: Figure S1B.

**Figure 4:**
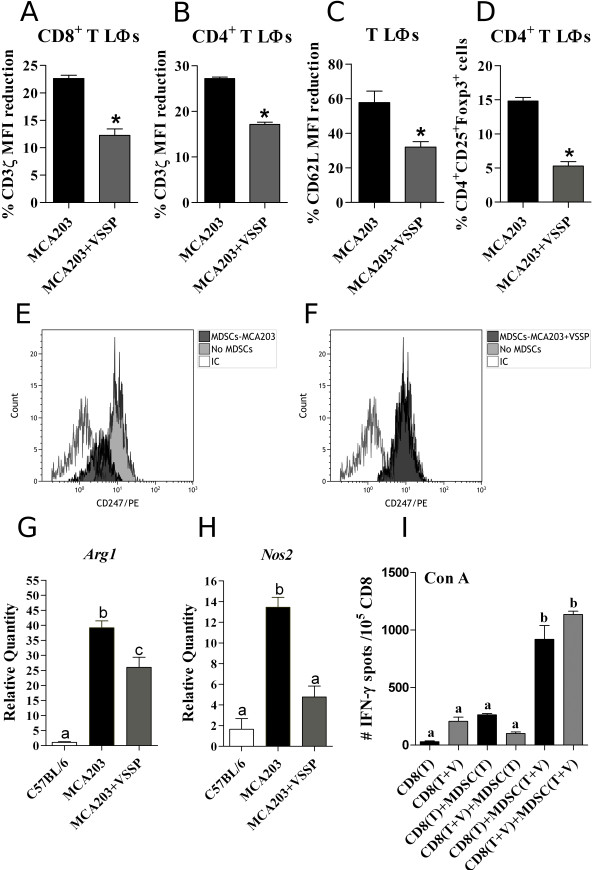
**Suppressive mechanisms of MDSCs are dampened by VSSP.** Mice were treated as described in Figure 2. FACS analyses of the down-regulation of CD3ζ chain on CD8^+^**(A)** and CD4^+^**(B)** T cells, as well as CD62L on T cells **(C)**, were performed in splenocytes from five individual mice per group. Data were normalized by the percentage of MDSCs in each mouse. The reduction in Tregs frequency caused by VSSP was further corroborated and resulted more obvious after the normalization procedure **(D)**. Statistical comparisons between groups were done with Student’s t test for CD62L expression and Tregs percentages, whereas Mann-Whitney’s U test was used to analyze down-regulation of CD3ζ chain. **(E-F)** MDSCs immunomagnetically enriched from the spleens of VSSP-injected or untreated MCA203 TB mice were cultured at 20% with splenocytes from OTI transgenic mice, in the presence of relevant peptide. Histograms show the expression of CD3ζ chain on CD8^+^ T cells specifically stimulated with SIINFEKL peptide in the presence or absence of MDSCs. Further characterization of MDSCs isolated from each experimental group was done by RT-PCR and the reduction of *Arg1***(G)** and *Nos*2 **(H)** gene expression is represented in bar graphs. Three replicates of CD11b^+^Gr1^+^ cells isolated from pools of three mice per group were included in the RT-PCR analysis. **(I)** The capacity of MDSCs to behave as APCs during Con A-stimulated IFN-γ production by CD8^+^ T cells was measured through ELISPOT assay. CD8^+^ T cells and CD11b^+^Gr1^+^ cells were isolated from TB mice, either treated or not with the adjuvant, and cocultured for 72 h in the presence of Con A mitogen. Graph indicates the mean ± SD of the number of IFN-γ spots per 10^5^ CD8^+^ T cells from one experiment representative of two (T: tumor and V: VSSP). **(G-I)** The multiple comparisons of mean values were executed with ANOVA and Tukey’s tests.

Among the negative effects of MDSCs on T lymphocytes, the down-regulation of both CD3ζ chain and the homing molecule CD62L has been widely described [35,36]. Thus, the expression of these molecules on T cells from MCA203 TB mice, inoculated or nor with VSSP, was determined. Notably, *in vivo* VSSP reduced the tumor-induced down-regulation of CD3ζ chain on both CD4^+^ and CD8^+^ T cells (Figure 4A and B). Likewise, down-regulation of CD62L on T cell populations was diminished in TB mice treated with VSSP (Figure 4C). Considering individually normalized data related to the percentage of MDSCs, again Tregs frequency was significantly lower in MCA203 TB mice injected with VSSP than in untreated TB mice (Figure 4D), reinforcing that VSSP could be reducing the capacity of tumor-induced MDSCs to expand Tregs.

To perform a more accurate analysis of MDSCs capacity to down-regulate CD3ζ chain on CD8^+^ T cells, OTI splenocytes were stimulated with SIINFEKL peptide in the presence of 20% MDSCs derived from different sources. As shown in Figure 4E, MDSCs isolated from MCA203 TB mice effectively down-regulated CD3ζ chain on antigen-specific CD8^+^ T cells (2.9 fold of reduction). In contrast, MDSCs derived from VSSP-inoculated TB mice were not able to down-regulate this key molecule for TCR signaling (Figure 4F). A further comparison by RT-PCR of CD11b^+^Gr1^+^ cells isolated from MCA203 TB mice, either inoculated or not with VSSP, evidenced a significant reduction of *Arg1* and *Nos2* gene expression as a consequence of VSSP treatment (Figure 4G and H). It has been extensively validated that ARG1 and NOS2 enzymes are key players for MDSCs suppressive capacity and, among other effects, contribute to CD3ζ chain down-regulation on T cells (revised in [14]).

Another feature of MDSCs is their impaired function as APCs. Thus, we wonder whether the capacity of MDSCs to behave as APCs could be enhanced by inoculating VSSP into MCA203 TB mice. Con A is a well-known mitogen that requires APCs to achieve an efficient activation of T cells [37]. Therefore, CD8^+^ T cells purified from either VSSP-inoculated or untreated MCA203 TB mice were stimulated with Con A in the presence of the corresponding MDSCs. MDSCs derived from untreated TB mice failed to stimulate Con A-induced production of IFN-γ by these effector cells, whereas MDSCs purified from VSSP-inoculated TB mice significantly increased the production of this cytokine by CD8^+^ T cells, regardless of the origin of these lymphocytes (Figure 4I). These results indicate that MDSCs from VSSP-treated TB mice could be functionally more differentiated to mature APCs.

### *In vitro* treatment of MDSCs with VSSP induces their differentiation and loss of suppressive activity

**Figure 5:**
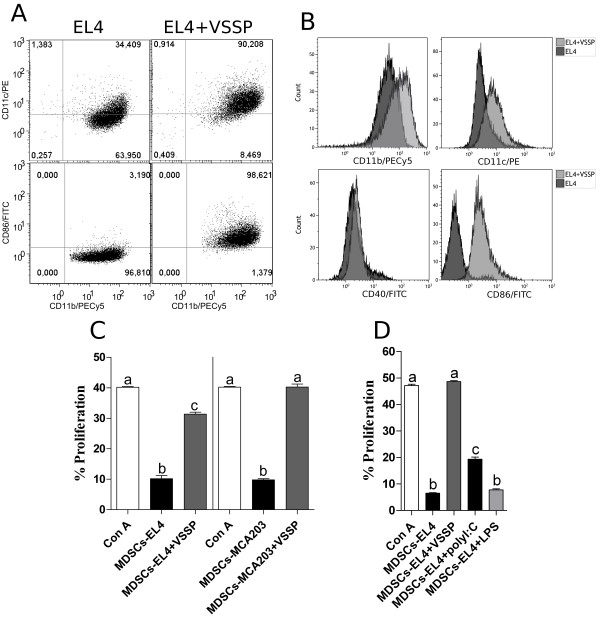
**VSSP induces differentiation of tumor-promoted MDSCs towards DCs *in vitro*.** Highly purified MDSCs from the spleen of EL4 TB mice were incubated for 24 h with VSSP. **(A-B)** Expression of CD11c, CD11b, CD40 and CD86 was evaluated by FACS within the CD11b^+^Gr1^+^ gate. **(C-D)** Inhibition of T cell proliferation was assessed by the culture of 40% *in vitro*-treated MDSCs with splenocytes from naive mice in the presence of Con A. **(C)** Effect of VSSP on the functional activity of MDSCs induced by EL4 and MCA203 tumors. **(D)** MDSCs from EL4 TB mice were also cultured *in vitro* with polyI:C or LPS and their suppressive activity was compared with VSSP-treated MDSCs. ANOVA and Tukey’s tests were employed for statistical comparison. These experiments were repeated three times with similar results.

Considering that *in vivo* administration of VSSP modifies tumor-induced MDSCs to display APCs functions, we hypothesized that probably the incubation of these MDSCs with the adjuvant *in vitro* will induce their differentiation to mature APCs. Based on our previous experiments in which VSSP inoculation *in vivo* promoted differentiation of adoptively transferred tumor-induced MDSCs to DCs and not to macrophages [24], we focused our analysis particularly on differentiation towards CD11c^+^ DCs *in vitro.* A more than 2 fold increase was detected in both, percentage and mean fluorescence intensity (MFI) of CD11c^+^ cells, within EL4-induced MDSCs treated *in vitro* with VSSP (*p* = 0.001 and *p* = 0.002, respectively) (Figure 5A and B). Furthermore, incubation with VSSP also augmented the expression of CD40 and CD86 molecules in these MDSCs, indicating a more mature stage of the cells (Figure 5A and B). A higher MFI associated to CD11b marker was seen additionally on VSSP-treated MDSCs. Similar results were obtained with MDSCs isolated from EG.7 and MCA203 TB mice (Additional file 3: Figure S3). Looking at the modification of the functional activity, we found that VSSP treatment *in vitro* significantly reduced the suppression of T cell proliferation mediated by MDSCs, isolated from these TB mice (Figure 5C).

The next question to address was whether another widely used adjuvant, polyI:C, has a VSSP-like effect on MDSCs differentiation to DCs. VSSP caused a higher increase than polyI:C in CD11b (3.18 vs 2.21 fold) and CD11c (3.02 vs 2.06 fold) molecules on tumor-induced MDSCs. Moreover, polyI:C did not significantly change the expression of Gr1 (1.1 fold) and CD40 (0.75 fold) whereas VSSP provoked near 2 fold increase in both markers (Additional file 4: Figure S4A and B). Accordingly, incubation of EL4-induced MDSCs with polyI:C caused a reduction in the suppressive capacity of these cells, but to a significantly lesser extent than VSSP (Figure 5D).

**Figure 6:**
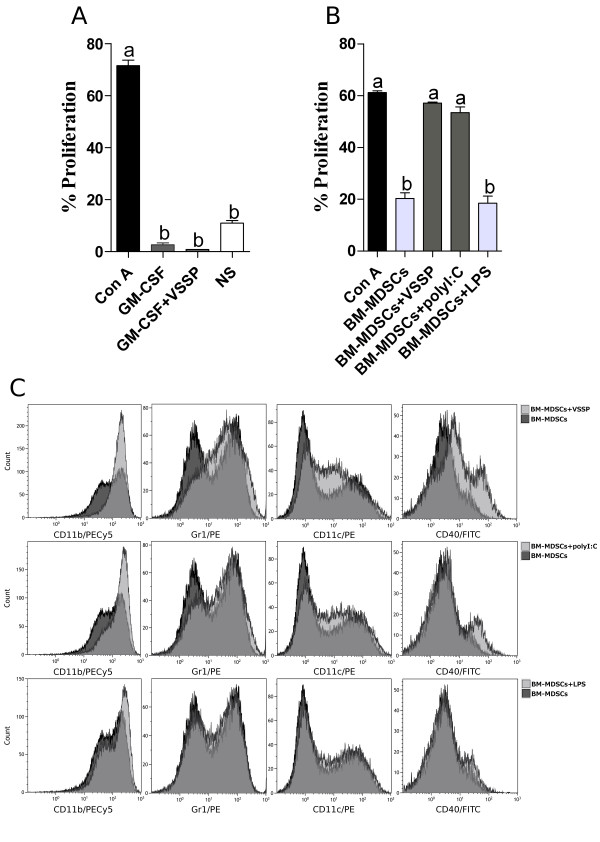
**Suppressive phenotype and function of BM-MDSCs are abolished after 24 h culture with VSSP. (A)** BM precursors were cultured with 40 ng/mL of GM-CSF for four days, in the presence or absence of VSSP. The capacity of these MDSCs (10% of the effector splenocytes) to inhibit Con A-induced proliferation of T cells was next evaluated. **(B-C)** BM-MDSCs differentiated *in vitro* only with GM-CSF were treated with VSSP, polyI:C or LPS for an additional 24 h. Afterward, the suppressive activity **(B)** and the phenotype within the CD11b^+^Gr1^+^ gate **(C)** were studied as mentioned above. Statistical differences in bar graphs were detected with ANOVA and Tukey’s tests. Two repetitions of this experiment were done with similar outcome.

To complete this study we tested the *in vitro* effect of VSSP on BM-MDSCs. When VSSP was added during differentiation of BM precursors with GM-CSF the inhibitory function of the resulting MDSCs was not changed (Figure 6A), although it promoted a phenotypic modification of these cells (Additional file 5: Figure S5). However, the treatment of already differentiated BM-MDSCs with VSSP effectively abrogated their suppressive activity (Figure 6B). Resembling the results obtained with tumor-induced splenic MDSCs, BM-MDSCs incubated with VSSP showed a higher expression of CD11c, CD11b, Gr1 and CD40 than untreated control cells (Figure 6C). Stimulation of BM-MDSCs with polyI:C caused a similar modification than VSSP in the former markers, except for CD40 which expression was not changed by polyI:C and increased 2 fold with VSSP (Figure 6C). The suppression of T cell proliferation by BM-MDSCs was also completely eliminated after the treatment with polyI:C, in contrast to the mild effect it provoked on tumor-induced MDSCs (Figure 6B).

Finally, since VSSP contains a TLR4 agonist, an additional comparison with bacterial lipopolysaccharide (LPS) was performed in these experimental settings. As shown in Additional file 4: Figure S4A and C, LPS treatment of tumor-induced MDSCs also caused a 2 fold increase in the expression of CD11b, Gr1 and CD11c molecules, similar to the effect of VSSP. However, LPS was unable to up-regulate maturation markers like CD40 in these MDSCs (Additional file 4: Figure S4C) and, more importantly, did not modify their suppressive activity (Figure 5D). Additionally, the incubation of BM-MDSCs with LPS did not significantly change the expression of CD11c, CD11b, Gr1 and CD40 molecules (Figure 6C) and, consequently, these cells retained their capacity to suppress the proliferation of T cells (Figure 6B). Taken together, these results suggest that the effect of VSSP on differentiation of MDSCs into DCs is not a common characteristic of any TLR4 agonist, but a particular property of VSSP.

### VSSP inhibits cross-presentation of TAA antigens by splenic CD11b^+^Gr1^+^ cells

**Figure 7:**
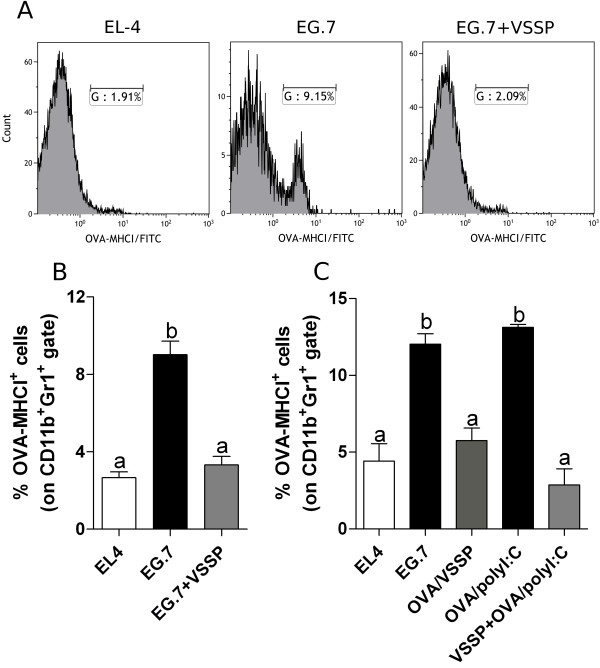
**Cross-presentation of TAA by splenic MDSCs is abrogated in VSSP-inoculated TB mice. (A-B)** EL4 and EG.7 TB mice were injected with VSSP and cross-presentation of the OVA peptide SIINFEKL was detected by FACS on splenic CD11b^+^Gr1^+^ cells, two days after the last dose of the adjuvant. **(A)** Histograms show the staining with the mAb specific for H-2K^b^-SIINFEKL peptide complexes on gated CD11b^+^Gr1^+^ cells for one representative mouse per group. **(B)** Mean ± SD of the percentages of MDSCs expressing OVA peptide bound to MHC I molecule (n = 9 to n = 15 individual mice per group belonging to three replicated experiments). **(C)** Mice with EG.7 tumors were vaccinated with OVA protein mixed with VSSP or polyI:C. Another group of TB mice was inoculated with VSSP and additionally received OVA/polyI:C vaccine. EL4 TB mice were included as negative controls. Cross-presentation of OVA antigen was analyzed as described above. These data are from two independent experiments. Statistical analyses were performed with ANOVA and Tukey’s tests.

Accordingly to previous findings that splenic CD11b^+^Gr1^+^ cells can cross-present tumor-antigens and tolerize specific CD8^+^ T lymphocytes [15], a similar experimental setting was selected to analyze whether VSSP could hamper this important process for tumor-induced immunosuppression (Additional file 1: Figure S1C). In fact, SIINFEKL peptide presented in H-2K^b^ molecules was detected with a specific Ab on splenic CD11b^+^Gr1^+^ cells from EG.7 TB mice (Figure 7A, B and C). Remarkably, the treatment of EG.7 TB mice with VSSP reduced to undetectable levels the cross-presentation of OVA, as a model tumor antigen, by splenic CD11b^+^Gr1^+^ cells, comparable to the values obtained for MDSCs isolated from EL4 TB mice, a control tumor lacking OVA expression (Figure 7A and B). Cross-presentation of OVA-derived SIINFEKL peptide by MDSCs subpopulations *in vivo* was also further analyzed. As shown in Additional file 6: Figure S6, both monocytic MDSCs (M-MDSCs, CD11b^+^Gr1^lo^) and polymorphonuclear MDSCs (PMN-MDSCs, CD11b^+^Gr1^hi^) obtained from the spleen of EG.7 TB mice cross-presented OVA protein. Interestingly, VSSP treatment inhibited cross-presentation of this TAA by both MDSCs subpopulations (Additional file 6: Figure S6).

**Figure 8:**
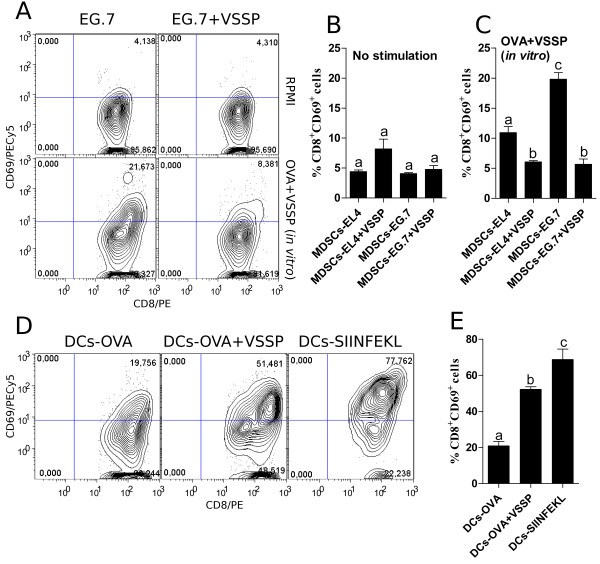
**Tolerogenic MDSCs incubated with VSSP are conditioned to cross-prime antigen-specific CD8**^**+ **^**T cells.** CD11b^+^Gr1^+^ cells isolated from EL4 and EG.7 TB mice, inoculated or not with VSSP, were treated *in vitro* with 10 μg/mL of both OVA and VSSP **(A and C)** or left untreated **(A and B)**. To assess the capacity of these cells to accomplish a detectable cross-priming, antigen-specific CD8^+^ T cells were isolated from OTI transgenic mice and cocultured at 1:1 ratio with MDSCs for 96 h. BM-DCs previously incubated with OVA, OVA and VSSP or pulsed with SIINFEKL peptide were used as controls **(D and E)**. Graphs show the percentage of CD69^+^ cells as a measure of the activation of antigen-specific CD8^+^ T cells. ANOVA and Tukey’s tests were used for statistical comparison of the groups’ mean. Two experiments with similar results were performed.

To analyze whether OVA cross-presentation by MDSCs conducted to tolerance of specific CD8^+^ T cells, OTI CD8^+^ T cells were cocultured with CD11b^+^Gr1^+^ cells isolated from EG.7 TB mice, inoculated or not with VSSP, in the absence of exogenous peptide. As expected for the suppressive function of MDSCs, CD11b^+^Gr1^+^ cells isolated from untreated mice bearing EG.7 tumors, that cross-presented OVA peptide *in vivo*, failed to activate antigen-specific CD8^+^ T cells, measured by the up-regulation of CD69 molecule (Figure 8A and B). Even though VSSP interferes with the suppressive mechanisms of MDSCs, in this experimental setting CD11b^+^Gr1^+^ cells obtained from VSSP-treated EG.7 TB mice did not stimulate OTI lymphocytes (Figure 8A and B). This apparent contradiction could be associated with the lack of specific peptide in MHC I complexes from these MDSCs, which is required to provide the “signal one” to CD8^+^ T cells. In fact, based on earlier results indicating that VSSP induces cross-presentation of OVA protein by DCs *in vitro*[27], we tested whether a similar mechanism could be functioning on MDSCs and this way we could supply the missing “signal one”. As shown in Figure 8A and C, CD11b^+^Gr1^+^ cells isolated from VSSP-injected mice, either bearing EL4 or EG.7 tumors, were unable to cross-present directly OVA protein in the presence of VSSP *in vitro*. These results suggest that VSSP is not capable to potentiate cross-presentation of OVA protein *in vitro* by MDSCs. Only EG.7-induced MDSCs, that cross-presented tumor-associated OVA peptide *in vivo*, achieved a moderate cross-priming of specific CD8^+^ T cells after incubation with OVA and VSSP *in vitro* (Figure 8A and C). Again the incubation of tolerogenic MDSCs with VSSP transformed these cells into APCs able to activate CD8^+^ T cells in an antigen-specific fashion, probably due to the promotion of a DCs-like phenotype with increased expression of co-stimulatory molecules that was demonstrated earlier in this work. As a positive control, BM-DCs induced a potent activation of CD8^+^ T cells as a consequence of *in vitro* culture with OVA and VSSP (Figure 8D and E).

Another relevant issue to address was whether administration of an OVA-containing vaccine could increase cross-presentation of this TAA by MDSCs and tolerize rather than activate tumor-specific CTLs (Additional file 1: Figure S1D-F). Vaccination with OVA adjuvated in VSSP inhibited cross-presentation of this antigen by MDSCs in EG.7 TB mice, whereas immunization with the same protein in polyI:C did not change the percentage of MDSCs expressing SIINFEKL peptide (Figure 7C). Noteworthy, concomitant VSSP administration also significantly inhibited cross-presentation of OVA antigen in EG.7 TB mice vaccinated with this protein adjuvated in polyI:C (Figure 7C). These results suggest that VSSP could be used to abrogate cross-presentation of TAA by MDSCs as a vaccine adjuvant or even employed as an immunomodulator together with other vaccines.

## Discussion

There is growing evidence in the literature indicating that the selection of a suitable adjuvant for cancer vaccines should pursue beyond the classical characteristics of an adjuvant for preventive vaccination. Thus, the additional property to overcome tumor-induced immunosuppression is of particular relevance. We have previously shown that a vaccine containing VSSP protects CTL responses in TB mice whereas DCs vaccination and polyI:C-based vaccine both generate reduced CTL responses in comparison to tumor-free mice [24]. In this work we evidenced that VSSP is also able to potentiate CTL responses induced by other vaccine in the immunosuppressive environment promoted by a tumor. As MDSCs play a key role on tumor-induced immunosuppression [14,38], the effect of this adjuvant on the suppressive mechanisms of MDSCs was evaluated. First we demonstrated that VSSP injection in MCA203 TB mice impairs the up-regulation of *Arg1* and *Nos2* gene expression observed in control MDSCs from untreated TB mice. Both, the depletion of L-arginine through ARG1 [39] and the production of peroxynitrite and hydrogen peroxide through the coordinated action of NOS2, ARG1 and NADPH Oxidase [14,40] could cause the down-regulation of CD3ζ chain. Accordingly, MDSCs from VSSP-treated TB mice were unable to down-regulate CD3ζ chain on antigen-specific CD8^+^ T cells *in vitro*. Moreover, splenic CD4^+^ and CD8^+^ T cells displayed higher expression of CD3ζ chain *in vivo*, as well as CD62L, in TB mice treated with VSSP, suggesting a smaller degree of impairment of their activation and functional activity.

Tregs play an important role also in tumor-induced immunosuppression and it has been described that MDSCs can further increase this regulatory population [16,17]. Our results indicate that expansion of CD11b^+^Gr1^+^ cells in TB mice, as caused by VSSP, is not necessarily connected to the augment of Tregs. In fact, VSSP administration reduced the population of splenic Tregs to levels observed in tumor-free mice, even though the percentage of MDSCs was 2 fold higher in comparison to untreated TB mice. In contrast, the inoculation of tumor-free mice with VSSP did not change the percentage of Tregs, indicating that there is not a direct effect of the adjuvant on these cells. These results strongly suggest that MDSCs from VSSP-treated TB mice possess a decreased capacity to expand Tregs, which could be linked among other factors to their lower expression of ARG1, enzyme that seems to be required for this process [17].

Several approaches for the pharmacological targeting of MDSCs have been studied in pre-clinical and clinical settings [14]. However, to our knowledge only docetaxel [20], paclitaxel [41] and all-trans retinoic acid [21,22] are able to induce their differentiation to mature myeloid cells. This is in our opinion the most desirable choice of interference with MDSCs function due to the concomitant increase in the number of APCs. In this work we showed that *in vitro* VSSP treatment of both tumor-induced MDSCs and BM-MDSCs was sufficient to increase not only CD11c marker, typically associated with DCs, but also the co-stimulatory molecules CD40 and CD86. Furthermore these MDSCs lost their suppressive activity after incubation with the adjuvant. This *in vitro* effect of VSSP connects with the capacity of splenic MDSCs, isolated from TB mice inoculated with the adjuvant, to potentiate the Con A-mediated stimulation of IFN-γ production by tumor-conditioned CD8^+^ T cells. The optimal stimulation of T cells caused by Con A requires APCs due to indirect cross-linking of the TCR [37].

Previous work by Shirota *et al.* established that CpG ODN, a TLR9 ligand, induces differentiation of CT26 tumor-induced MDSCs into F4/80^+^ macrophages, which display M1 phenotype and possess a significant cytotoxic activity against CT26 tumor cells [23]. In contrast, incubation of BM-MDSCs with the combination of the TLR4 agonist LPS and IFN-γ increases NO production, potentiates the suppressive activity of MDSCs and impairs DCs development from this immature population [42]. For polyI:C, which triggers TLR3 signaling, the effect is less clear. Zoglmeier *et al.* have shown that this adjuvant only provokes the conversion of tumor-induced MDSCs into macrophages in the presence of IFN-α produced by plasmacytoid DCs [43]. In this work we found that polyI:C also increased the percentage of CD11c^+^ cells on tumor-induced MDSCs and BM-MDSCs, but to a lesser extent than VSSP. Noteworthy, polyI:C was unable to up-regulate the maturation markers on both kinds of MDSCs whereas VSSP significantly increased it. In agreement with Greifenberg *et al.*[42], LPS was incapable to drive a complete differentiation of MDSCs to mature APCs in our experiments, and CD11b^+^Gr1^+^ cells retained their suppressive function. It is currently accepted that MDSCs require a first signal inducing their expansion and a second signal (like TLR signaling) to fully activate their suppressive mechanisms [44]. All this emerging evidence of the effect of different TLR ligands on the suppressive function and differentiation status of MDSCs reveals their complex interaction, even though the signaling pathways for TLRs are quite limited.

It has been demonstrated that the spleen is a key organ for tumor-induced tolerance, in which MDSCs cross-present TAA to antigen-specific CD8^+^ T cells in a tolerogenic fashion [15]. We showed in this work that VSSP was capable to abrogate cross-presentation of tumor antigens by splenic MDSCs, whereas polyI:C was unable to modify this process, indicating a particular characteristic of VSSP. Furthermore, concomitant administration of VSSP reduced cross-presentation of a model TAA by splenic MDSCs derived from TB mice vaccinated with this antigen adjuvated in polyI:C. To our knowledge this is the first report of the capacity of an immunomodulator to hamper this important mechanism for the tolerization of tumor-specific T cells. The mechanisms leading to antigen cross-presentation by MDSCs are poorly understood. Our results demonstrated that VSSP inhibits TAA cross-presentation by the two main subpopulations of MDSCs, which suggests an effect of the adjuvant on the mechanism of antigen cross-presentation itself, and cannot be explain solely by the shift in the relative amount of M-MDSCs and PMN-MDSCs. In addition, our previous work indicates that VSSP administration significantly reduce the migration of MDSCs towards the tumor site [24], which may help to explain the inhibition of TAA capture and processing by these cells. Nonetheless, there are other pathways employed by splenic MDSCs to capture tumor antigens that should be addressed in VSSP-treated mice. Amongst these pathways, a partial involvement of the uptake of tumor exosomes on TAA cross-presentation has been demonstrated [15,45]. The results of this study also suggest that, in contrast to DCs, MDSCs are unable to directly cross-present protein antigens in the presence of VSSP *in vitro*, an otherwise efficient stimulus for DCs-mediated cross-priming of CD8^+^ T cells.

The modulation caused by VSSP on several mechanisms associated with tumor-induced immunosuppression, mediated by MDSCs and Tregs, could ultimately contribute to the observed potentiation of tumor-specific CTL responses within the tumor site. In line with these results, it has been reported that MDSCs within the tumor microenvironment are also more differentiated to mature APCs in TB mice treated with VSSP [24]. These evidences suggest that, in VSSP-treated mice, the tumor microenvironment is more permissive to the antitumor effector functions of TILs. In fact, VSSP-based vaccines inhibit tumor growth in MCA203 and EG.7 TB mice [24].

## Conclusions

The present work was designed to investigate the immunomodulatory mechanisms of VSSP in TB hosts. In fact, VSSP treatment not only prevented up-regulation of *Arg1* and *Nos2* on tumor-induced MDSCs but also reduced MDSCs-mediated down-regulation of CD3ζ chain on antigen-specific CD8^+^ T cells. More importantly, VSSP abrogated TAA cross-presentation by MDSCs and induced their differentiation on mature DCs. Furthermore, TB mice treated with VSSP showed lower percentages of Tregs and more efficient CTL responses. Altogether these results validate the use of VSSP as immunomodulator, either as a cancer vaccine adjuvant or in combination strategies with other immunotherapies.

## Methods

### Mice

C57BL/6 mice (8 to 12-week-old) were purchased from the Center for Laboratory Animal Production (CENPALAB, Havana, Cuba) and OTI TCR transgenic mice were obtained from Charles River Laboratories (Wilmington, MA). All mice were maintained in the animal house of the Center of Molecular Immunology (CIM, Havana, Cuba). The experiments were performed in accordance with institutional guidelines from the CIM animal care and use committee.

### Reagents and cell lines

VSSP was prepared at CIM by hydrophobic conjugation of the GM3 ganglioside with outer membrane vesicles from *Neisseria meningitidis* strain 385 (Finlay Institute, Havana, Cuba), as described elsewhere [26]. Chicken OVA grade VII, Con A from *Canavalia ensiformes*, LPS from *Escherichia coli*, PMA, Ionomycin from *Streptomyces conglobatus* and polyI:C were purchased from Sigma-Aldrich (Saint Louis, MO). OVA_257-264_ peptide (SIINFEKL) was synthetized at the Center for Genetic Engineering and Biotechnology (CIGB, Havana, Cuba) and CFSE was obtained from Invitrogen (Paisley, UK). Recombinant murine GM-CSF was purchased from PeproTech (NJ, USA). EL4 and EG.7 (EL4 cell line transfected with the gene encoding for OVA) lymphomas, and MCA203 sarcoma are derived from C57BL/6 mice (H-2^b^) and were kindly provided by Dr. Vincenzo Bronte. The metastatic clone of Lewis lung carcinoma cell line (3LL-D122) also syngeneic for C57BL/6 strain was obtained from the American Type Culture Collection (ATCC, Manassas, VA).

### FACS analysis

Cells were stained with specific antibodies or control isotypes using conventional protocols. The following anti-mouse antibodies were used for FACS analysis of MDSCs: CD11b/PECy5, Gr1/PE, Gr1/APC, CD62L/FITC, F4/80/FITC, CD11c/PE, and MHC CI OVA peptide/Biotin from eBiosciences (San Diego, CA); as well as CD124/PE, CD40/FITC, CD86/FITC, I^A^-I^E^/FITC, Ly6C/FITC and Ly6G/PE that were purchased from BD Biosciences (San Jose, CA). Lymphocytes characterization was performed with the next specific antibodies: CD8/PE and CD8/Biotin, CD69/PECy5, CD62L/FITC, CD247/PE, CD4/PE, CD4/PECy5, CD25/PECy5, Foxp3/Biotin, IFN-γ/FITC, IL-4/PE and IL-17/PE, all from eBiosciences. FITC-conjugated Streptavidin was purchased also from eBiosciences. In all cases, cells were previously incubated with anti-mouse FcγR 2.4G2 ascites (HB-197; ATCC) to reduce the non-specific binding. For intracellular staining, cells were fixed and permeabilized, according to the manufacturer’s protocol, with either Foxp3 Staining Buffer Set or IC Fixation and 10X Permeabilization Buffers from eBiosciences. Cells were acquired using both FACScan (BD Biosciences) and Gallios (Beckman Coulter, Miami, FL) flow cytometers and analyzed with Kaluza 1.2 (Beckman Coulter) and FlowJo 5.7.2 (Tree Star Inc., USA) softwares.

### Generation of BM-derived MDSCs and DCs

BM cells were harvested from femurs and tibias of C57BL/6 mice. For DCs preparation, the cells were cultured for 6 days with 20 ng/mL of GM-CSF as described elsewhere [46]. BM-MDSCs were obtained in Petri dishes after a 4 days incubation of 2.5×10^5^ BM cells with 40 ng/mL of GM-CSF. Additionally, VSSP was added during MDSCs differentiation at a concentration of 10 μg/mL.

### Immunization protocols

To evaluate the immunomodulatory properties of VSSP, C57BL/6 mice were s.c. challenged on day 0 with 1×10^6^ cells of MCA203, EL4 or EG.7 tumor lines. VSSP (200 μg/mice) was next administered on days 11, 12 and 18 to MCA203 TB mice. Likewise, mice with EL4 and EG.7 tumors were inoculated on days 4, 5 and 11 with the adjuvant. In some experiments mice received a vaccine containing OVA protein (1 mg/mice) mixed with either VSSP or polyI:C (100 μg/mice). PolyI:C was given on days 13, 14 and 15 for MCA203 tumor model and 4, 5 and 6 in EG.7 TB mice. All experiments were performed on days 22 and 13 for MCA203 and EL4 (or EG.7) tumor models, respectively. Immunization protocols were summarized and depicted in Additional file 1: Figure S1.

### Modulation of Th differentiation and CTL responses by VSSP

The analysis of tumor-specific Th responses was performed by 96 h stimulation of 4×10^5^ splenocytes, from VSSP-treated or untreated MCA203 TB mice, with 2×10^4^ BM-DCs. These BM-DCs were previously pulsed with tumor lysates obtained from 10×10^6^ cells of MCA203 and 3LL-D122 tumor lines. On the last 10 h, PMA (50 ng/mL) and Ionomycin (500 ng/mL) were added to the culture, together with the Golgi blocking reagent Monensin (as recommended by the manufacturer, eBiosciences). IFN-γ, IL-17 and IL-4 were measured by intracellular staining as describe above.

To evaluate the *in vivo* antigen-specific CTL response in vaccinated TB mice, splenocytes from naive mice were differentially labeled (5 min at 37°C) with CFSE. The cells stained with CFSE^high^ (5 μM) were used as targets and pulsed with SIINFEKL peptide (1 μM; 90 min at 37°C and 5% CO_2_), whereas the cells labeled with CFSE^low^ (0.33 μM) were kept unpulsed and served as internal control. After extensive washing to remove free peptide, target and control cells were mixed at 1:1 proportion and coinjected i.v. into vaccinated mice. Sixteen hours later, inguinal draining lymph nodes were harvested and the total events corresponding to both fluorescent intensities (CFSE^low^ and CFSE^high^) were determined by FACS. The percentage of specific lysis was calculated as: 100 - [(CFSE^high^/CFSE^low^) × 100].

### MDSCs isolation and *in vitro* differentiation to APCs

Spleens from TB mice, treated or not with VSSP, were harvested and single-cell suspensions prepared. MDSCs were isolated using magnetic microbeads conjugated with rat anti-mouse/human CD11b mAb (Miltenyi Biotec, Bergisch Gladbach, Germany), following the manufacturer’s instructions. Purity of the cell population was evaluated by FACS and exceeded 95%. More than 96% of isolated CD11b^+^ cells expressed Gr1 marker, which excluded any relevant contamination with conventional DCs or macrophages (data not shown).

To assess differentiation of MDSCs into APCs, 1×10^6^ splenic CD11b^+^Gr1^+^ cells or BM-MDSCs were cultured for 24 h in 6 well plates (BD Falcon, Oxford, UK) with VSSP (10 μg/mL), polyI:C (30 μg/mL), LPS (1 μg/mL), or left untreated. Molecules associated with DCs lineage, as well as markers of mature APCs, were detected by FACS within the CD11b^+^Gr1^+^ gate. Suppressive capacity of these cells was compared as indicated below.

### *In vitro* MDSCs-mediated suppression assay

Splenocytes (6×10^5^) from naive mice were stained with 2 μM of CFSE and cocultured with splenic CD11b^+^Gr1^+^ cells or BM-MDSCs, at different effector to suppressor ratios, in 96-well flat bottom plates (BD Falcon). T cells were stimulated by the addition of 2 μg/mL of Con A. Proliferation was measured by CFSE dilution after 96 h of culture.

### Down-regulation of CD3ζ chain by MDSCs

The down-regulation of CD3ζ chain caused by MDSCs was analyzed by culturing 6×10^5^ splenocytes from OTI transgenic mice with 1.2×10^5^ MDSCs, isolated from VSSP-treated or untreated MCA203 TB mice, in 96-well flat bottom plates (BD Falcon). OTI CD8^+^ cells were stimulated with 1 μM of SIINFEKL peptide for 72 h. The expression of CD3ζ chain on these CD8^+^ cells was analyzed by intracellular staining using the anti-mouse CD247/PE antibody. In addition, the down-regulation of CD3ζ and CD62L on splenic T cells was detected *in vivo* in mice with MCA203 tumors, treated or not with VSSP. The percentage of MFI reduction for CD3ζ and CD62L molecules was calculated as indicated in the formula: (MFI_PBS_–MFI_MCA203 or MCA203 + VSSP_)/MFI_PBS_ * 100. Afterward these data were normalized by the percentage of MDSCs in each mouse, in order to study the *in vivo* function of MDSCs at equivalent amounts.

### Real-time PCR

Total RNA extraction and real-time analysis were performed as previously described [24]. Data analyses were done with SDS 2.3 software.

### Restoration of MDSCs as functional APCs

The activity of MDSCs as functional APCs was evaluated through their capacity to potentiate Con A-induced stimulation of CD8^+^ T cells. IFN-γ secretion was measured in a classical ELISPOT assay in which cellulose-ester membrane microplates (Millipore, Milan, Italy) were coated with R4-6A2 mAb (BD Biosciences). Both CD8^+^ cells and MDSCs were positively selected from the spleens of TB mice using magnetic microbeads (Miltenyi Biotec) and following the manufacturer’s instructions. Purified CD8^+^ T cells (5×10^4^) were next stimulated for 72 h with 2 μg/mL of Con A in the presence of 2×10^4^ CD11b^+^Gr1^+^ cells. Plates were washed extensively, and spots were visualized with biotin-conjugated mAb XMG1.2 (BD Biosciences), alkaline phosphatase-conjugated goat anti-biotin antibody (Vector Laboratories, Peterborough, U.K.) and AP Substrate Kit (Bio-Rad Laboratories, Hempstead, U.K.). The number of spots was counted in triplicate and calculated using an automatic ELISPOT counter (ELISPOT Reader System ELRIFL04; AID, Straßberg, Germany).

### Functional analysis of TILs

MCA203 tumors from untreated or VSSP-inoculated mice were extracted on day 22 and TILs were isolated with microbeads conjugated with either anti-CD90 or anti-CD8 mAbs. To evaluate the tumor-specific response of CD8^+^ TILs, isolated CD8^+^ T cells (5×10^4^) were stimulated for 72 h with 1×10^5^ MCA203 cells, or MB16F10 negative control, both previously treated with IFN-α to increase MHC I expression. Non-specific stimulation of TILs with 2 μg/mL of Con A mitogen was additionally performed during 72 h. IFN-γ production was detected by ELISPOT assay as described above.

### Cross-presentation of TAA by MDSCs

Mice bearing EG.7 tumors inoculated with VSSP alone, and either OVA/VSSP or OVA/polyI:C vaccines, were euthanized and splenocytes stained with mAbs specific for CD11b, Gr1 and SIINFEKL peptide bound to H-2K^b^. Splenocytes from EL4 TB mice served as negative controls for the expression of OVA antigen.

For assessing the capacity of splenic MDSCs to achieve an effective cross-priming, antigen-specific CD8^+^ T cells were isolated from OTI transgenic mice by negative selection using CD8a^+^ T Cell Isolation Kit II (Miltenyi Biotec) and used as effector cells. Splenic MDSCs isolated from EG.7 and EL4 TB mice, inoculated or not with VSSP, were cultured at 1×10^6^ cells per well with 10 μg/mL of both OVA and VSSP for 24 h, or left untreated. Afterward, MDSCs were washed and cocultured at 1:1 ratio with 1×10^5^ OTI CD8^+^ cells for 96 h in 96-well flat bottom plates (BD Falcon). BM-DCs previously incubated with OVA, OVA and VSSP or pulsed with SIINFEKL peptide were used as controls. As a measure of CD8^+^ T cell activation, the expression of CD69 molecule was detected by FACS.

### Statistics

Equality of variances was analyzed with Bartlett’s test and Kolmogorov-Smirnov test was used to verify normal distribution of data. Comparisons of CD62L expression on T cells, IFN-γ secretion from TILs and changes in CD11c molecule on MDSCs *in vitro* were performed with Student’s *t* test (2-tailed), whereas the data regarding the down-regulation of CD3ζ chain *in vivo* was analyzed with Mann-Whitney’s U test. Statistical significance in the comparison of Th differentiation was tested by Kruskal-Wallis and Dunn’s non-parametric tests. All other statistical analyses were performed with one-way ANOVA and Tukey’s test for pairwise comparison, using SPSS 16.0 software (SPSS, Chicago, IL). The statistically significant differences (p < 0.05) between more than two groups were always represented within the graphs by diverse letters.

## Abbreviations

MDSCs: Myeloid-derived suppressor cells; M-MDSCs: Monocytic MDSCs; PMN-MDSCs: Polymorphonuclear MDSCs; TAA: Tumor-associated antigens; TILs: Tumor-infiltrating lymphocytes; VSSP: Very small size proteoliposomes; TB: Tumor-bearing; ARG: Arginase; NOS: Nitric oxide synthase.

## Competing interests

The authors declare that they have no competing interests.

## Authors’ contributions

Conceived and designed the experiments: AF, LEF and CM. Performed the experiments: AF, LO, RA, AH and JR. Analyzed the data: AF, LO and CM. Wrote the paper: AF, LEF and CM. All authors read and approved the final manuscript.

## Supplementary Material

Additional file 1: Figure S1 Schematic representation of the treatment protocols to evaluate the immunomodulatory properties of VSSP. MCA203 tumors were implanted s.c. in the flank of C57BL/6 mice, on day 0, and a group of TB mice additionally received three doses of VSSP on days 11, 12 and 18. **(A)** To evaluate *in vivo* CTL responses potentiated by VSSP, MCA203 TB mice inoculated with VSSP were further vaccinated with OVA adjuvated in polyI:C on days 13, 14 and 15. **(B)** Spleens from MCA203 TB mice, treated or not with VSSP, were harvested on day 22 and the percentage, inhibitory function and characterization of MDSCs suppressive mechanisms were addressed. This protocol was used likewise to determine percentages of Tregs, as well as CD3ζ chain and CD62L expression on T lymphocytes. Splenocytes from these mice were also tested to study the modulation caused by VSSP on tumor-induced polarization of Th cells specific for TAA. **(C-F)** C57BL/6 mice were s.c. challenged with EL4 or EG.7 tumor cells on day 0. **(C)** EG.7 TB mice, as well as control mice with EL4 tumors, were inoculated on days 4, 5 and 11 with VSSP, to measure on day 13 the effect of the adjuvant on cross-presentation of TAA by splenic MDSCs. **(D-E)** To assess whether administration of an OVA-containing vaccine could change cross-presentation of this TAA by MDSCs, EG.7 TB mice were immunized with OVA protein mixed with either VSSP **(D)** or polyI:C **(E)**. VSSP-containing vaccine was administered on days 4, 5 and 11 **(D)** whereas the vaccine employing polyI:C was inoculated on days 4, 5 and 6 **(E)**. **(F)** Another group of EG.7 TB mice was vaccinated with OVA adjuvated in polyI:C on days 4, 5 and 6 and additionally received three doses of VSSP (days 4, 5 and 11). On day 13, the modulatory effect of VSSP on cross-presentation of this TAA (OVA) by splenic MDSCs was evaluated on vaccinated EG.7 TB mice. Click here for file

Additional file 2: Figure S2 Effect of VSSP treatment on the phenotype and suppressive activity of MCA203-induced splenic MDSCs. MCA203 tumors were s.c. grown in C57BL/6 mice and both tumor-free and TB mice were inoculated three times with VSSP. Another group of animals with tumors remained untreated. **(A)** CD11b^+^Gr1^+^ cells were enriched from pools of spleens by magnetic microbeads and stained with the indicated antibodies. From top to bottom panel: FACS profile of MDSCs from VSSP-treated tumor-free mice, MCA203 TB mice, and mice with MCA203 tumors inoculated with VSSP. **(B)** To evaluate the inhibition of CD8^+^ T cell responses, 20% MDSCs were incubated for 72 h with 4×10^5^ SIINFEKL-pulsed splenocytes isolated from tumor-free mice previously inoculated with OVA mixed with polyI:C. The IFN-γ production was detected by a classical ELISPOT assay and the number of IFN-γ spots per 10^5^ CD8^+^ T cells is indicated in the graph. **(C)** 2×10^4^ effector splenocytes were cocultured with 1×10^5^ SIINFEKL-pulsed EL4 target cells or non-pulsed controls, in the presence of 20% MDSCs. Target cells were pre-treated with 25 μg/mL of Mitomycin C to avoid proliferation. After 92 h of culture, supernatans were recovered and the release of lactate de-hydrogenase (LDH) measured as recommended by the manufacturer (Roche Diagnostics, Indianapolis, IN). Mean percentage of specific lysis from triplicate wells was determined by the formula: *%* cytotoxicity = [(experimental - spontaneous LDH release)/(maximum - spontaneous LDH release)] × 100. **(B-C)** Statistically significant differences were detected with ANOVA and Tukey’s tests. These results are representative of at least two experiments. Click here for file

Additional file 3: Figure S3 Differentiation of MDSCs isolated from different TB mice due to incubation with VSSP *in vitro***.** CD11b^+^Gr1^+^ cells isolated from the spleen of MCA203 **(A)** and EG.7 **(B)** TB mice were cultured with 10 μg/mL VSSP for 24 h, or left untreated. Histograms show the expression of the molecules CD11c, CD11b, CD40 and CD86 detected by FACS within the gate of CD11b^+^Gr1^+^ cells. Two repetitions of this experiment were done with similar outcome. Click here for file

Additional file 4: Figure S4 Comparative effect of VSSP, polyI:C and LPS on differentiation of EL4-induced MDSCs towards DCs *in vitro*.** (A)** VSSP (10 μg/mL), **(B)** polyI:C (30 μg/mL) and **(C)** LPS (1 μg/mL) were added *in vitro* to EL4-induced MDSCs for 24 h. Afterwards, cells were washed and stained with anti-mouse Abs specific for CD11c, CD11b, Gr1 and CD40 markers. Untreated tumor-induced MDSCs were included as control of immature population. Histograms are referred to the gate of CD11b^+^Gr1^+^ cells. The results shown in this figure are representative of two similar experiments. Click here for file

Additional file 5: Figure S5 Phenotypic characterization of MDSCs generated *in vitro* from BM precursors, in the presence or absence of VSSP. BM-precursors were cultured during 4 days with 40 ng/mL of GM-CSF. VSSP (10 μg/mL) was added through the complete time of culture, together with GM-CSF. The pseudocolor graphs show the staining with CD11b, Gr1, F4/80, Ly6G and IL-4Rα on MDSCs generated by both culture conditions. This experiment was done twice and comparable effect was observed. Click here for file

Additional file 6: Figure S6 Effect of VSSP on cross-presentation of TAA by PMN-MDSCs and M-MDSCs. Mice bearing EL4 or EG.7 tumors were injected with VSSP and cross-presentation of the OVA peptide SIINFEKL was detected, two days after the last VSSP injection, on splenic PMN-MDSCs (CD11b^+^Gr1^hi^) and M-MDSCs (CD11b^+^Gr1^lo^). **(A)** Gaiting strategy followed to design MDSCs subpopulations is shown for one representative mouse per group. Typical histograms corresponding to the staining with the mAb specific for MHC I-SIINFEKL complexes on each MDSCs subpopulation are also depicted. **(B-C)** Mean ± SD of the percentage of PMN-MDSCs **(B)** and M-MDSCs **(C)** expressing OVA peptide bound to MHC I molecules (n = 9 mice per group belonging to two different experiments). Diverse letters indicate statistically significant differences by ANOVA and Tukey’s tests. Click here for file
