# Supplementary figures and images for: Very small size proteoliposomes abrogate cross-presentation of tumor antigens by myeloid-derived suppressor cells and induce their differentiation to dendritic cells
Source: J Immunother Cancer. 2014 Mar 11;2:5. doi: 10.1186/2051-1426-2-5 (PMC4019907; doi:10.1186/2051-1426-2-5)

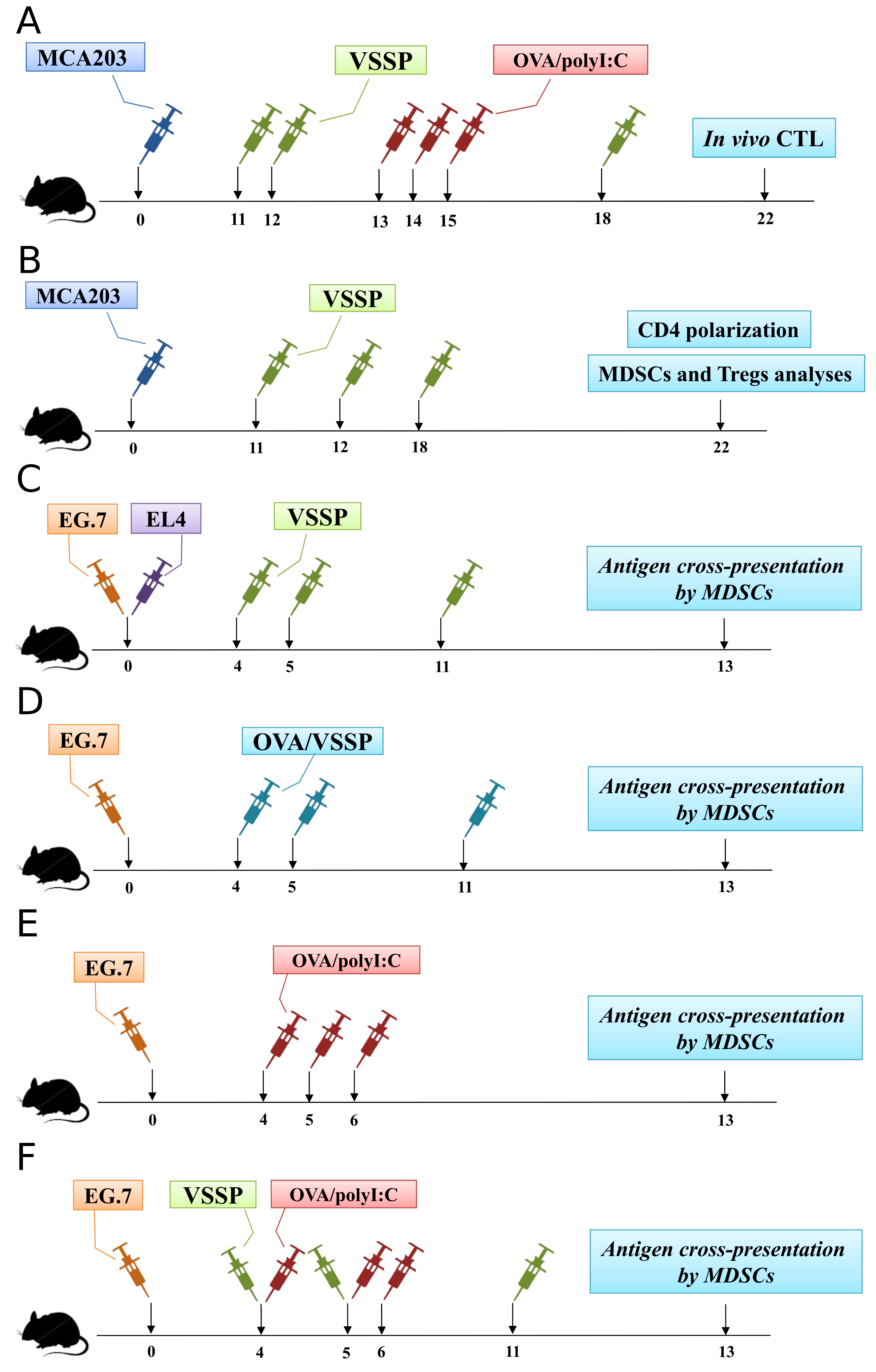

Supplement: Additional file 1: Figure S1 — Schematic representation of the treatment protocols to evaluate the immunomodulatory properties of VSSP. MCA203 tumors were implanted s.c. in the flank of C57BL/6 mice, on day 0, and a group of TB mice additionally received three doses of VSSP on days 11, 12 and 18. (A) To evaluate in vivo CTL responses potentiated by VSSP, MCA203 TB mice inoculated with VSSP were further vaccinated with OVA adjuvated in polyI:C on days 13, 14 and 15. (B) Spleens from MCA203 TB mice, treated or not with VSSP, were harvested on day 22 and the percentage, inhibitory function and characterization of MDSCs suppressive mechanisms were addressed. This protocol was used likewise to determine percentages of Tregs, as well as CD3ζ chain and CD62L expression on T lymphocytes. Splenocytes from these mice were also tested to study the modulation caused by VSSP on tumor-induced polarization of Th cells specific for TAA. (C-F) C57BL/6 mice were s.c. challenged with EL4 or EG.7 tumor cells on day 0. (C) EG.7 TB mice, as well as control mice with EL4 tumors, were inoculated on days 4, 5 and 11 with VSSP, to measure on day 13 the effect of the adjuvant on cross-presentation of TAA by splenic MDSCs. (D-E) To assess whether administration of an OVA-containing vaccine could change cross-presentation of this TAA by MDSCs, EG.7 TB mice were immunized with OVA protein mixed with either VSSP (D) or polyI:C (E). VSSP-containing vaccine was administered on days 4, 5 and 11 (D) whereas the vaccine employing polyI:C was inoculated on days 4, 5 and 6 (E). (F) Another group of EG.7 TB mice was vaccinated with OVA adjuvated in polyI:C on days 4, 5 and 6 and additionally received three doses of VSSP (days 4, 5 and 11). On day 13, the modulatory effect of VSSP on cross-presentation of this TAA (OVA) by splenic MDSCs was evaluated on vaccinated EG.7 TB mice. [file 2051-1426-2-5-S1.tiff]

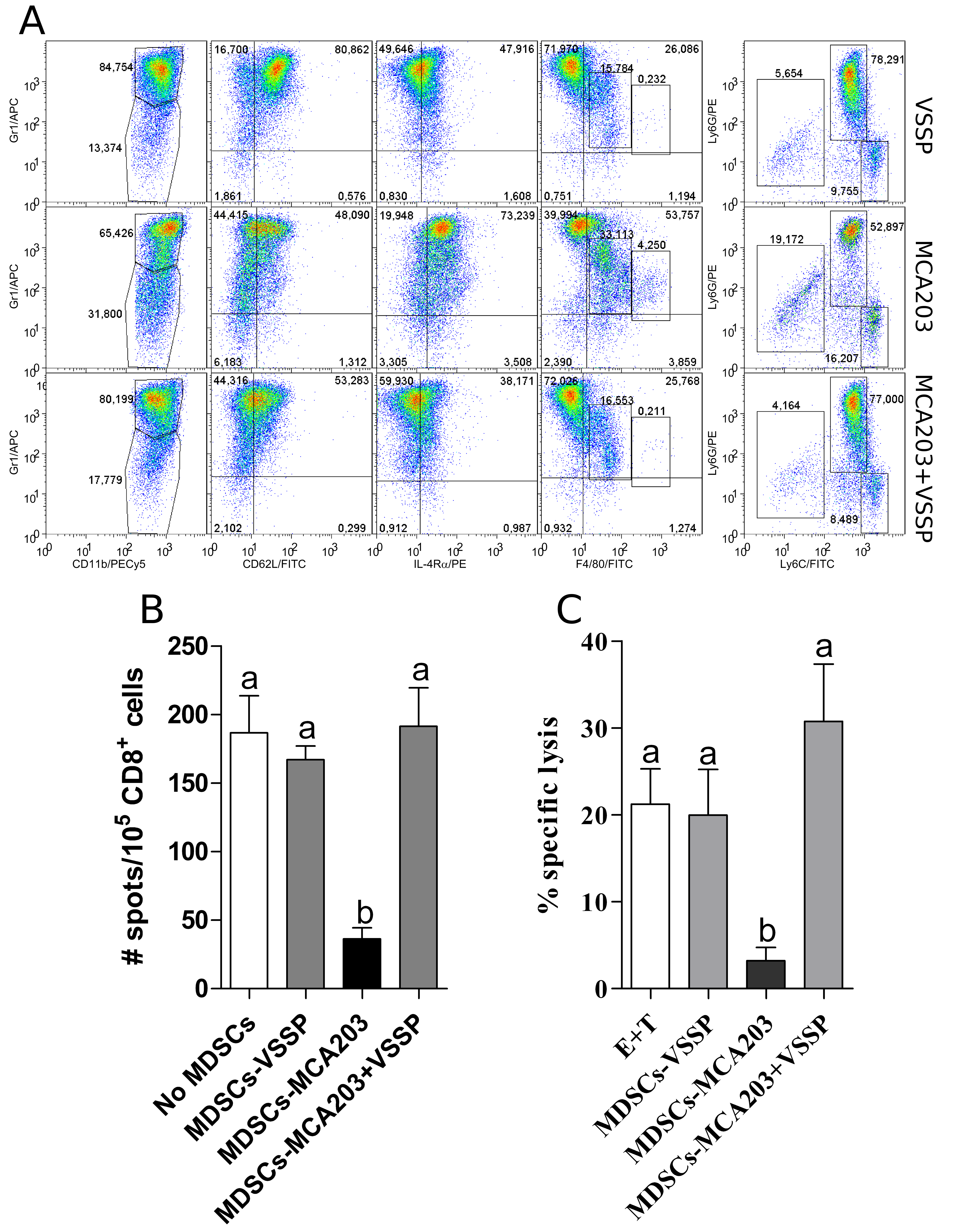

Supplement: Additional file 2: Figure S2 — Effect of VSSP treatment on the phenotype and suppressive activity of MCA203-induced splenic MDSCs. MCA203 tumors were s.c. grown in C57BL/6 mice and both tumor-free and TB mice were inoculated three times with VSSP. Another group of animals with tumors remained untreated. (A) CD11b+Gr1+ cells were enriched from pools of spleens by magnetic microbeads and stained with the indicated antibodies. From top to bottom panel: FACS profile of MDSCs from VSSP-treated tumor-free mice, MCA203 TB mice, and mice with MCA203 tumors inoculated with VSSP. (B) To evaluate the inhibition of CD8+ T cell responses, 20% MDSCs were incubated for 72 h with 4×10^5^ SIINFEKL-pulsed splenocytes isolated from tumor-free mice previously inoculated with OVA mixed with polyI:C. The IFN-γ production was detected by a classical ELISPOT assay and the number of IFN-γ spots per 105 CD8+ T cells is indicated in the graph. (C) 2×10^4^ effector splenocytes were cocultured with 1×10^5^ SIINFEKL-pulsed EL4 target cells or non-pulsed controls, in the presence of 20% MDSCs. Target cells were pre-treated with 25 μg/mL of Mitomycin C to avoid proliferation. After 92 h of culture, supernatans were recovered and the release of lactate de-hydrogenase (LDH) measured as recommended by the manufacturer (Roche Diagnostics, Indianapolis, IN). Mean percentage of specific lysis from triplicate wells was determined by the formula: % cytotoxicity = [(experimental - spontaneous LDH release)/(maximum - spontaneous LDH release)] × 100. (B-C) Statistically significant differences were detected with ANOVA and Tukey’s tests. These results are representative of at least two experiments. [file 2051-1426-2-5-S2.tiff]

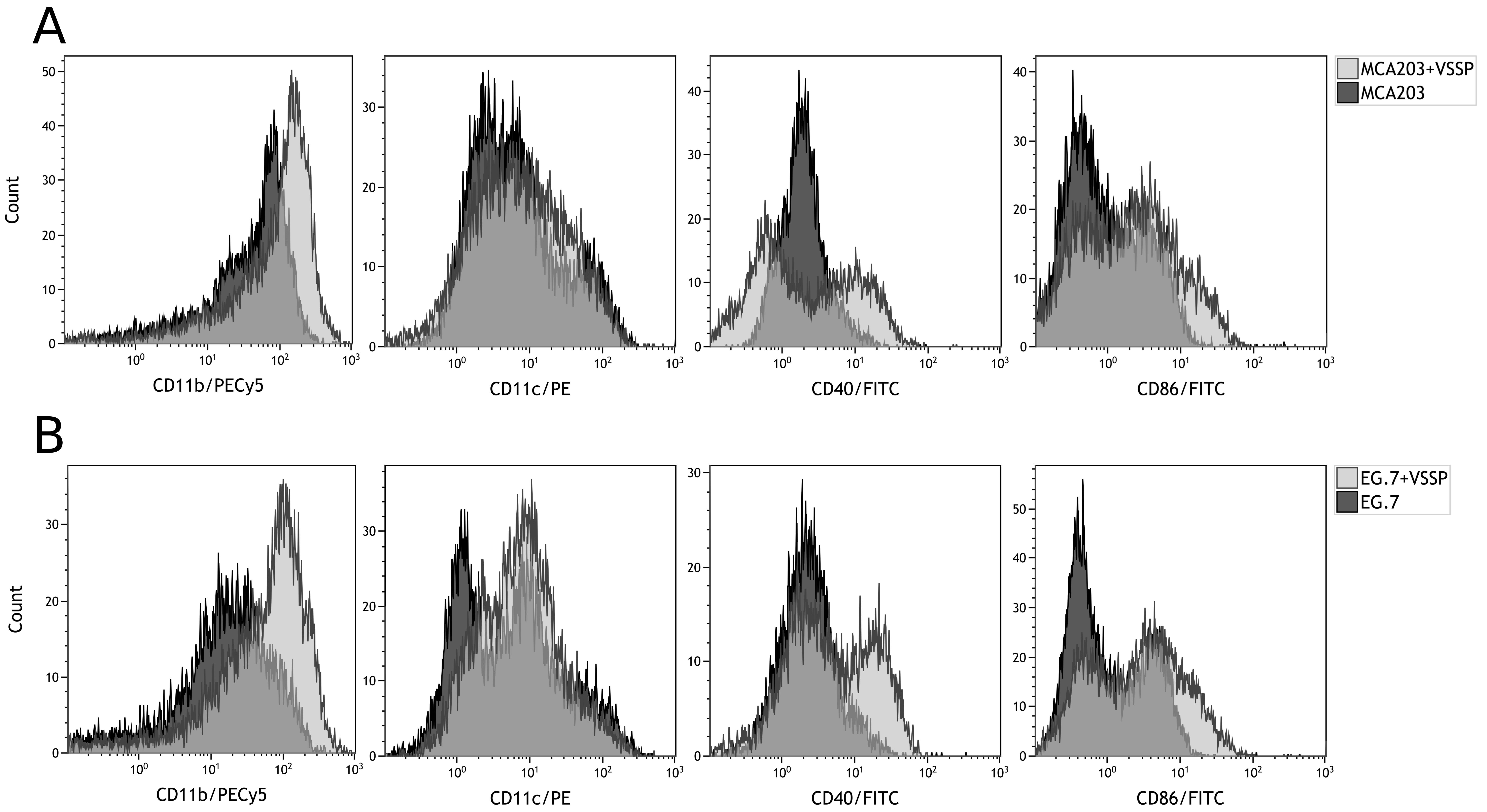

Supplement: Additional file 3: Figure S3 — Differentiation of MDSCs isolated from different TB mice due to incubation with VSSP in vitro. CD11b+Gr1+ cells isolated from the spleen of MCA203 (A) and EG.7 (B) TB mice were cultured with 10 μg/mL VSSP for 24 h, or left untreated. Histograms show the expression of the molecules CD11c, CD11b, CD40 and CD86 detected by FACS within the gate of CD11b+Gr1+ cells. Two repetitions of this experiment were done with similar outcome. [file 2051-1426-2-5-S3.tiff]

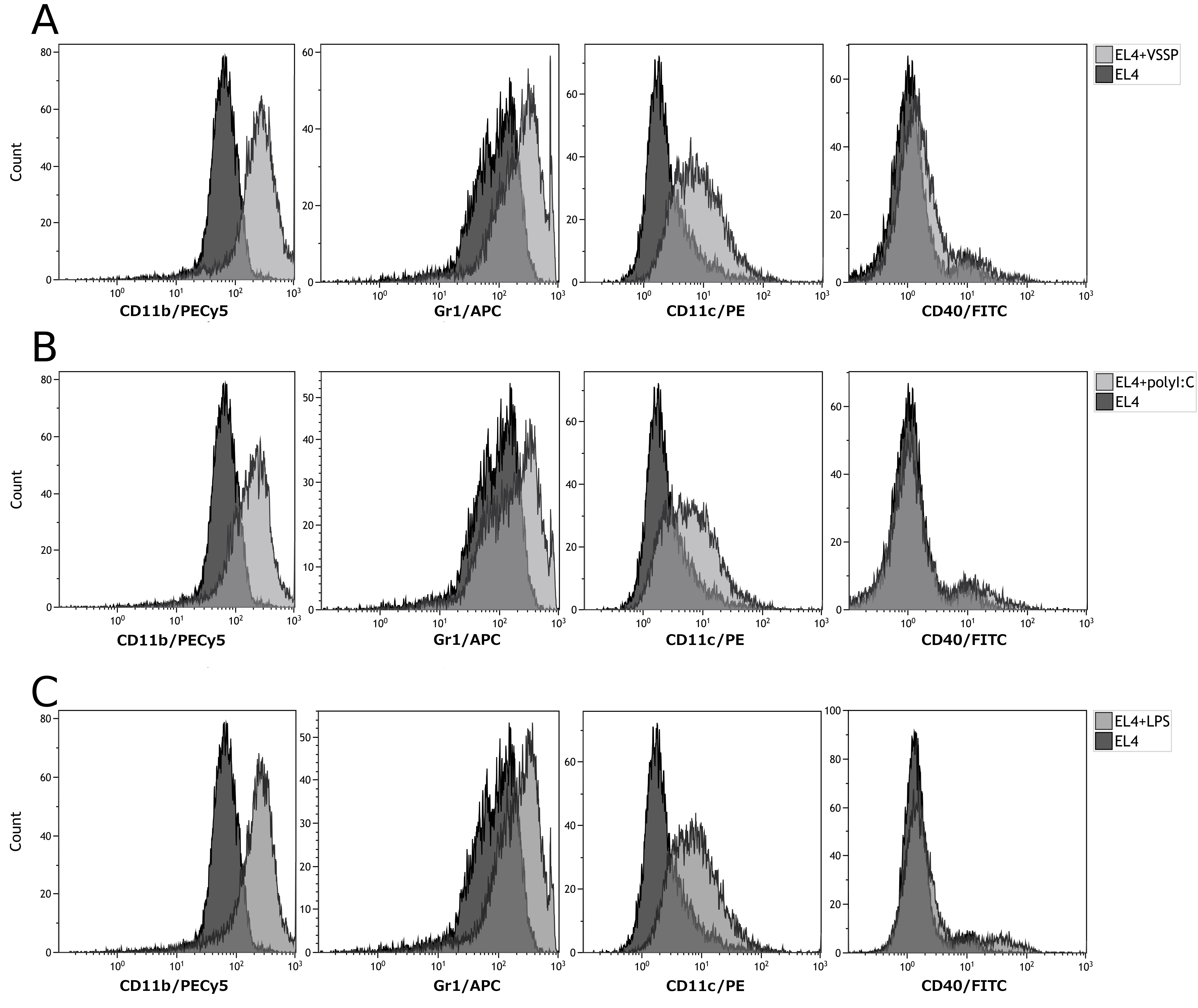

Supplement: Additional file 4: Figure S4 — Comparative effect of VSSP, polyI:C and LPS on differentiation of EL4-induced MDSCs towards DCs in vitro. (A) VSSP (10 μg/mL), (B) polyI:C (30 μg/mL) and (C) LPS (1 μg/mL) were added in vitro to EL4-induced MDSCs for 24 h. Afterwards, cells were washed and stained with anti-mouse Abs specific for CD11c, CD11b, Gr1 and CD40 markers. Untreated tumor-induced MDSCs were included as control of immature population. Histograms are referred to the gate of CD11b+Gr1+ cells. The results shown in this figure are representative of two similar experiments. [file 2051-1426-2-5-S4.tiff]

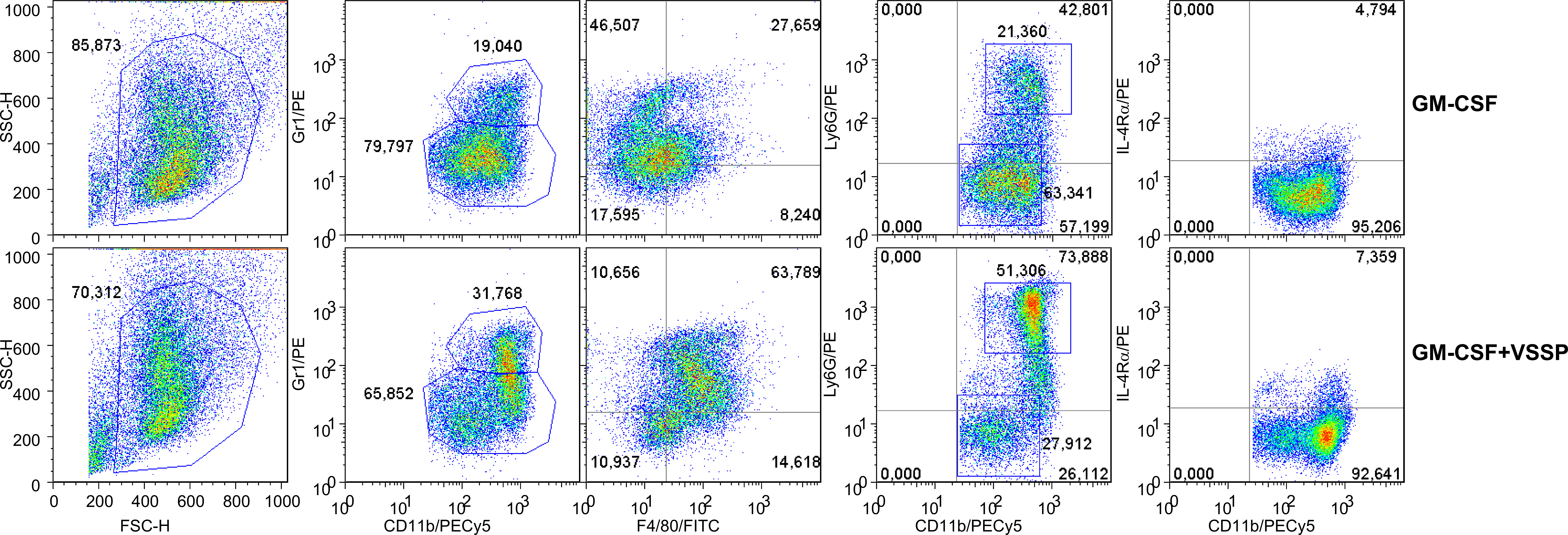

Supplement: Additional file 5: Figure S5 — Phenotypic characterization of MDSCs generated in vitro from BM precursors, in the presence or absence of VSSP. BM-precursors were cultured during 4 days with 40 ng/mL of GM-CSF. VSSP (10 μg/mL) was added through the complete time of culture, together with GM-CSF. The pseudocolor graphs show the staining with CD11b, Gr1, F4/80, Ly6G and IL-4Rα on MDSCs generated by both culture conditions. This experiment was done twice and comparable effect was observed. [file 2051-1426-2-5-S5.tiff]

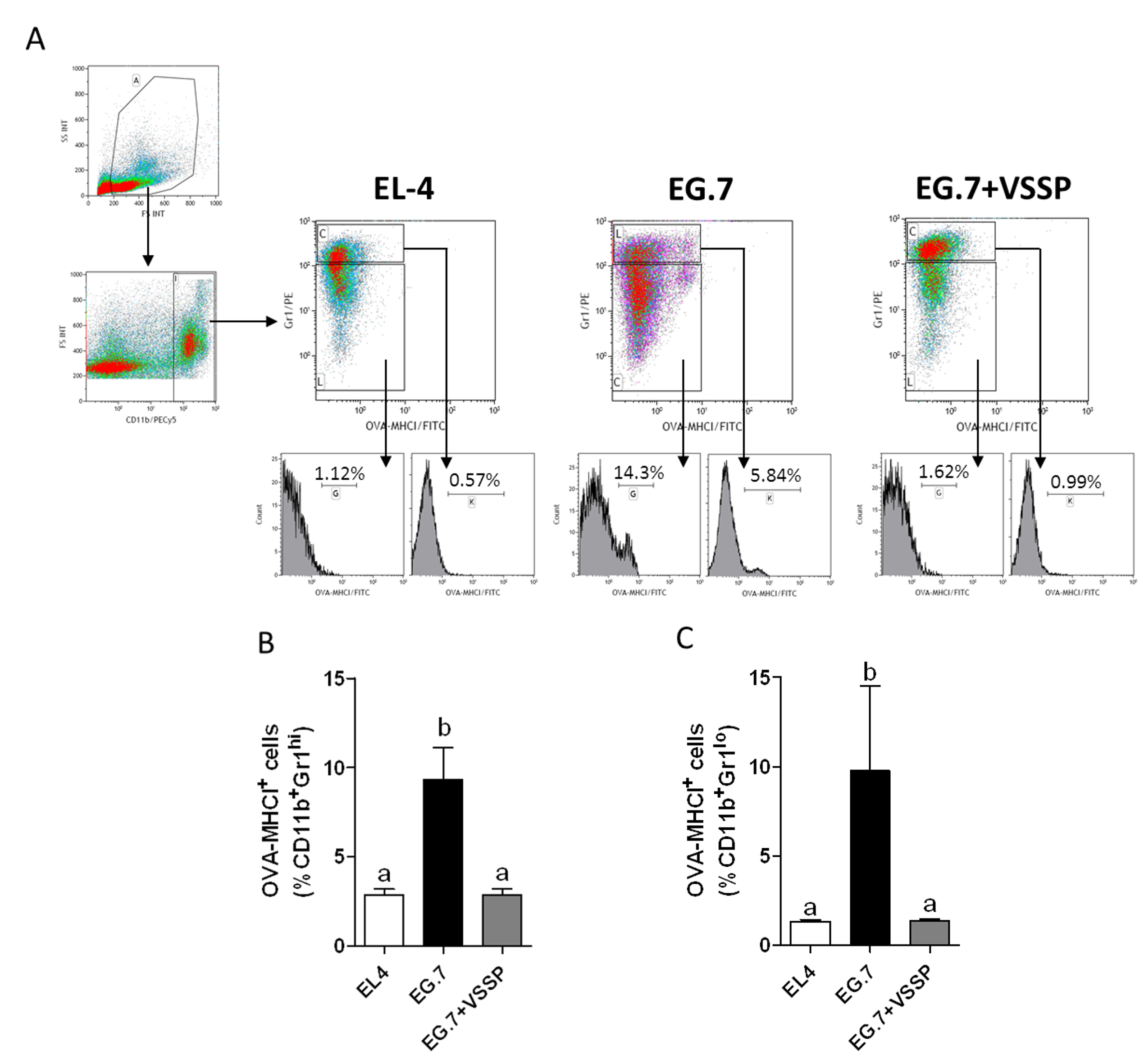

Supplement: Additional file 6: Figure S6 — Effect of VSSP on cross-presentation of TAA by PMN-MDSCs and M-MDSCs. Mice bearing EL4 or EG.7 tumors were injected with VSSP and cross-presentation of the OVA peptide SIINFEKL was detected, two days after the last VSSP injection, on splenic PMN-MDSCs (CD11b+Gr1hi) and M-MDSCs (CD11b+Gr1lo). (A) Gaiting strategy followed to design MDSCs subpopulations is shown for one representative mouse per group. Typical histograms corresponding to the staining with the mAb specific for MHC I-SIINFEKL complexes on each MDSCs subpopulation are also depicted. (B-C) Mean ± SD of the percentage of PMN-MDSCs (B) and M-MDSCs (C) expressing OVA peptide bound to MHC I molecules (n = 9 mice per group belonging to two different experiments). Diverse letters indicate statistically significant differences by ANOVA and Tukey’s tests. [file 2051-1426-2-5-S6.tiff]
